# Design and Analysis of a Silicon-Based Pattern Reconfigurable Antenna Employing an Active Element Pattern Method

**DOI:** 10.3390/mi8010011

**Published:** 2017-01-04

**Authors:** Ke Han, Zhongliang Deng, Xubing Guo

**Affiliations:** School of Electronic Engineering, Beijing University of Posts and Telecommunications, Beijing 100876, China; hanke@bupt.edu.cn (K.H.); dengzl@bupt.edu.cn (Z.D.)

**Keywords:** silicon-based, RF MEMS pattern reconfigurable antenna, Ka-band, RF MEMS switch, active element pattern method

## Abstract

In this paper, a silicon-based radio frequency micro-electromechanical systems (RF MEMS) pattern reconfigurable antenna for a Ka-band application was designed, analyzed, fabricated, and measured. The proposed antenna can steer the beam among three radiating patterns (with main lobe directions of −20°, 0°, and +20° approximately) at 35 GHz by switching RF MEMS operating modes. The antenna has a low profile with a small size of 3.7 mm × 4.4 mm × 0.4 mm, and consists of one driven patch, four parasitic patches, two assistant patches, and two RF MEMS switches. The active element pattern method integrated with signal flow diagram was employed to analyze the performances of the proposed antenna. Comparing the measured results with analytical and simulated ones, good agreements are obtained.

## 1. Introduction

Pattern reconfigurable antennas received considerable attention owing to its attractive performance, as they can switch radiating patterns while keeping other operating parameters unchanged, such as operating frequency and polarization. In spacecraft, satellite, and missile applications, antenna constraints include weight, size, cost, and aerodynamic profile. Thus, the implementation of a pattern reconfigurable antenna with a low profile can alleviate those constraints. To date, many pattern reconfigurable antennas [1,2,3,4,5] have been developed, and reconfigurable antennas are commonly implemented using variodes [6,7] and PIN diodes [1,2]. Compared with the variodes, PIN diodes, and other technologies, radio frequency (RF) micro-electromechanical systems (MEMS) switches possess many attractive advantages, such as high linearity, high quality factors, and almost no Please check the sections highlight in yellow in “the whole text” as we have made little modification. direct current (DC) power consumption [8]. Many reconfigurable antennas have been developed by employing MEMS switches [9,10,11,12]. However, most of those reconfigurable antennas operate at a low frequency and do not have process consistence, namely, the RF MEMS switches were mounted on circuitry after the antenna patch was implemented, instead of the integrated manufacture of the antenna patch and the RF MEMS switches. In addition, with the various superiorities such as a wide bandwidth, a compact device structure, and a high data throughput capacity, devices in a Ka-band have many advantages.

A pattern reconfigurable antenna that consisted of a driven patch (active element) and two assistant radiated patches (passive elements) was designed [6], but the proposed method to design and analyze the assistant patches was severely dependent on full-wave simulation, which is time-consuming. A beam-steering antenna was designed in [1], and the antenna was comprised of active patch and passive patch elements, but the analysis of the passive patch elements was rough and had no quantified calculations. In [13] an antenna using parasitic coupling was designed, but the analysis of the parasitic coupling function was insufficient, and the antenna only had simulated results. The active element pattern method integrated with the signal flow diagram needs to be shown to be effective in analyzing the passive antenna patches (parasitic coupling) [14,15]. The active element pattern of an element is defined as its radiation pattern when all other elements terminate in matched loads [16], and an antenna can be fully described by the its active element pattern and scattering parameters. This method is employed to design and analyze the proposed RF MEMS pattern reconfigurable antenna in this study.

In this paper, a pattern reconfigurable antenna operating at 35 GHz is proposed by employing RF MEMS switches. By changing the two RF MEMS switches operating modes, the proposed antenna can switch among four different kinds of operating states and obtain three kinds of reconfigurable patterns (because two operating modes possess the same pattern). The proposed pattern reconfigurable antenna is analyzed using an active element pattern method and a signal flow diagram. Comparing the calculated and simulated results with the measured ones, good agreement is acquired.

This paper is divided into five sections: Section 2 illustrates the design of the pattern reconfigurable antenna, Section 3 analyzes the operating mechanism of the proposed pattern reconfigurable antenna, Section 4 displays the measurement and results, and Section 5 summarizes the paper.

## 2. Design of the Pattern Reconfigurable Antenna

### 2.1. Antenna Design

**Figure 1:**
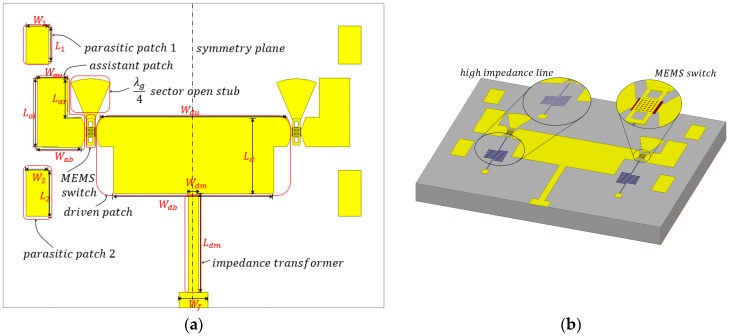
Proposed pattern reconfigurable antenna. (**a**) The configuration of proposed antenna. (**b**) The 3D view of the proposed antenna.

A pattern reconfigurable is designed and its structure is illustrated in Figure 1, the close-ups shown in Figure 1b are the RF MEMS switch and its DC actuating circuit. The geometry configurations of Figure 1a are shown in Table 1. The antenna consists of one rectangle driven patch radiator, four rectangle parasitic patches, two assistant patches, and two RF MEMS switches. The RF MEMS switch was terminated by a λ*_g_*/4 sector open stub and a high resistivity bias line. Parastic patch 1 and 2 are used for extending the operating bandwidth. The proposed pattern reconfigurable antenna patches and RF MEMS switches were all fabricated on a high resistivity silicon substrate with a thickness of 400 μm and a dielectric constant of 11.9.

By changing the mode of the RF MEMS switches, the antenna is capable of switching among four operating modes (00, 01, 10, 11; the “0” represents the up state, and the “1” stands for the down state) and achieving three pattern reconfigurable states in the *yoz* plane (φ = 90°) accordingly, because two of the operating modes are at the same pattern reconfigurable state. According to the measurement results of the fabricated pattern reconfigurable antenna, the three reconfigurable radiating patterns at operating frequency 35 GHz were obtained, i.e., left (approximately −20°), middle (approximately 0°), and right (approximately +20°), respectively. The detailed results of the reconfigurable radiating pattern main lobe direction and its associated RF MEMS switches operating modes are shown in Table 2.

### 2.2. RF MEMS Switch Design

**Figure 2:**
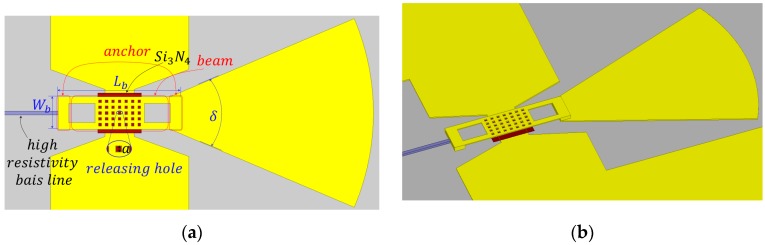
Designed RF MEMS switch. (**a**) Top view. (**b**) 3D view.

The reconfigurations of the proposed antenna are realized by controlling the modes of RF MEMS switches; thus, the performance of RF MEMS switch is critical for the overall system. The thickness of the RF MEMS switch beam is 1 μm, and the air gap between the beam and the signal line that connects the derived patch and assistant patch is 1.5 μm. The other pivotal size is shown in Table 3. The top view and 3D view of the designed RF MEMS switch are illustrated in Figure 2.

As shown in Figure 2, the RF MEMS switch is terminated by a λ*_g_*/4 sector open stub and a high resistivity bias line, which is used to apply the direct current. To alleviate the influences on return loss and resonant frequency, the λ*_g_*/4 sector open stub is employed, which has many advantages such as miniaturization of struction and the convenience of connection. The dielectric shown in Figure 2a,b is Si_3_N_4_ with a thickness of 0.15 μm, and it is used to separate the beam and the signal line when the air gap is 0 μm. By actuating the RF MEMS switches using the direct current, the gap between the beam and the signal line varies from 0 to 1.5 μm (i.e., down state to up state). The down or up state determinate whether the assistant patch is connected to the driven patch, i.e., when the switch is in the down state (the gap is 0 μm), the assistant patch is detached from the driven patch, and vice versa.

According to the simulated results, the isolation of the proposed RF MEMS switch reaches 20 dB, and the insertion loss is 0.35 dB at operating frequency 35 GHz, respectively. The performance of the antenna can therefore be guaranteed. When the position of the RF MEMS switch beam is pulled to the point (2/3)*g*_0_, the increase of the restoring force is exceeded by the increase of the electrostatic force. This leads to a rapid drop-down of the RF MEMS beam, and the actuating voltage reaches a maximum. The actuating voltage can be calculated by

(1) $$V_{p}=\sqrt{\frac{2k_{e}}{\varepsilon_{0}W_{b}L_{d}}\frac{g_{0}}{3}{\left(\frac{2g_{0}}{3}+\frac{t_{e}}{\varepsilon_{r}}\right)}^{2}}$$

where ε_0_ is the dielectric constant of free space, ε*_r_* is the relative dielectric constant of dielectric Si_3_N_4_, *g*_0_ is the gap between the RF MEMS switch beam and the signal line, *k_e_* is the elastic coefficient of the beam, *t_e_* is the thickness of the beam, and *W_b_* and *L_d_* are the width and length of the beam, respectively. The actuating voltage is approximately 7.5 V calculated by Equation (1), but the measured voltage is actually 20.8 V. The measured actuating voltage is more than twice the calculated value, the main reasons for this phenomenon being the incomplete releasing of the polyimide and the inhomogeneity of the polyimide thickness. If the manufacture process has good release and flatness, the actuating voltage will be close to the theoretical value. The quality factor [8] of the RF MEMS switch is *Q* = [4ρ*t_e_*^2^*E*^−1/2^/μ(*W_b_L_d_*)^2^]*g*_0_^3^ ≈ 1. Thus, the pull in time of the RF MEMS switch is *ts* ≈ (27*V*_p_^2^)/(4ω_0_*QV_s_*^2^) ≈ 12.6 μs, where *E* is Young’s modulus, μ is the air viscosity coefficient between the RF MEMS beam and the Si_3_N_4_ dielectric, and Ω_0_ is the mechanical resonant frequency, respectively.

## 3. Theory Analysis of Pattern Reconfigurations

**Figure 3:**
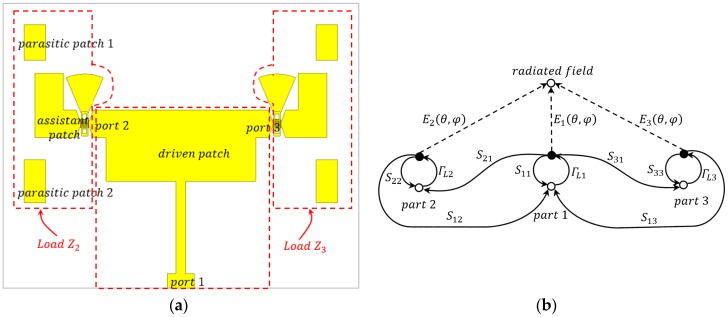
Divided parts of the antenna and its signal flow diagram. (**a**) Divided parts of the antenna. (**b**) Equivalent signal flow diagram of the proposed pattern reconfigurable antenna.

An antenna array can be fully described by its scattering parameters and the active element pattern of each radiating element [16]. The proposed pattern reconfigurable antenna in this paper can be viewed as the degenerated antenna array shown in Figure 3a. (The whole structure of the reconfigurable antenna can be divided into three parts shown in Figure 3a, i.e., the left part, middle part, and right part. These three parts constitute an antenna array with three elements. However, the three elements in this array are not identical; both the left and right parts did not equip a separate microstrip feed line. Therefore, the reconfigurable antenna is equivalent to a degenerate antenna array.) Thus, the active element pattern, the scattering parameters, and the signal flow diagram method can be employed to analyze the proposed antenna appropriately. The active element pattern of an element is defined as its radiation pattern when all other elements are terminated in matched loads [16]. In this paper, a method combining an active element pattern and a signal flow diagram [17], is employed to analyze the pattern reconfigurations of the antenna-based RF MEMS switches.

The proposed antenna can be divided into three parts. As shown in Figure 3a, the driven patch and the feed line comprise Part 1, and Part 2 (namely load *Z*_2_) consists of an assistant patch, a RF MEMS switch, parasitic patch 1, and parasitic patch 2, and the composition of Part 3 (namely load *Z*_3_) is the same as Part 2. The parasitic patch in Part 2 and Part 3 are used to slightly tune the frequency of the overall antenna structure and extend the operating bandwidth. Without any loss of analysis precision, the parasitic patches are involved in loads *Z*_2_ and *Z*_3_, as shown in Figure 3. Moreover, the port associated with each part is marked in Figure 3a as well.

The proposed antenna can be modeled as an equivalent scattering parameter matrix *S_D_*, which consists of a regular scattering parameter matrix *S_r_* and the electric field intensity matrix *E* of active element pattern. The equivalent scattering parameter matrix *S_D_* is defined as

(2) $$S_{D}=\left(\begin{array}{cc} S_{r} & E^{T}\\ E & 0 \end{array}\right)$$

and the electric field intensity matrix *E* is

(3) $$E=\left(\begin{array}{ccc} E_{1}\left(\theta ,\varphi \right) & E_{2}\left(\theta ,\varphi \right) & E_{3}\left(\theta ,\varphi \right) \end{array}\right)$$

where ***E****_i_*(θ, φ) is the electric field vector of each active element pattern, and *E^T^* is the transpose matrix of *E*.

As shown in Figure 3, the driven patch (part 1) is terminated by load *Z*_2_ and load *Z*_3_. The regular scattering matrix *S_r_* includes three ports, and *S*_32_ and *S*_23_ are all approximately equal to zero because the couplings between load *Z*_2_ and load *Z*_3_ can be neglected. According to the symmetry of the designed antenna overall structure, *S*_13_ = *S*_12_ and *S*_31_ = *S*_21_, respectively. Thus, *S_r_* is defined as

(4) $$S_{r}=\left(\begin{array}{ccc} S_{11} & S_{12} & S_{12}\\ S_{21} & S_{22} & 0\\ S_{21} & 0 & S_{33} \end{array}\right).$$

The signal flow diagram of the proposed antenna is shown in Figure 3b, all of those parameters are extracted from a full wave simulation. The Γ*_i_* is the reflection coefficient of Port No. *i*. Using the active element pattern and Mason rules, the return loss *RL* and gain *G(*θ, φ) of the proposed antenna are calculated as follows:

(5) $$RL=20\log\left(\frac{M}{P}\right)$$

(6) $$G\left(\theta ,\varphi \right)=20\log\frac{N}{P\sqrt{\sum_{n=1}^{3}{\left|I_{n}e^{j\varphi_{n}}\right|}^{2}}}$$

where $I_{n}e^{j\varphi_{n}}$is the signal current applied to Ports *n*, *M*, *N*, and *P*, which are defined as follows:

(7) $$\left\{ \begin{array}{l} M=S_{11}-S_{11}\Gamma_{L2}S_{22}-S_{11}\Gamma_{L3}S_{33}\\ +S_{11}\Gamma_{L2}S_{22}\Gamma_{L3}S_{33}+S_{12}^{2}\Gamma_{L2}-S_{12}^{2}\Gamma_{L2}\Gamma_{L3}S_{33}\\ +S_{13}^{2}\Gamma_{L3}-S_{13}^{2}\Gamma_{L2}\Gamma_{L3}S_{22}\\ N=E_{1}\left(\theta ,\varphi \right)\left(1-\Gamma_{L2}S_{22}-\Gamma_{L3}S_{33}+\Gamma_{L3}S_{33}\Gamma_{L2}S_{22}\right)\\ +E_{2}\left(\theta ,\varphi \right)S_{12}\Gamma_{L2}\left(1-\Gamma_{L3}S_{33}\right)\\ +E_{3}\left(\theta ,\varphi \right)S_{13}\Gamma_{L3}\left(1-\Gamma_{L2}S_{22}\right)\\ P=1+S_{11}\Gamma_{L1}\left[\Gamma_{L3}S_{33}+\Gamma_{L2}S_{22}-\Gamma_{L2}S_{22}\Gamma_{L3}S_{33}\right]+\Gamma_{L2}S_{22}\Gamma_{L3}S_{33}\\ -\Gamma_{L2}S_{12}^{2}\Gamma_{L1}-\Gamma_{L3}S_{13}^{2}\Gamma_{L1}-S_{12}^{2}\Gamma_{L2}\Gamma_{L3}S_{13}\Gamma_{L1}\\ -\Gamma_{L2}S_{22}-\Gamma_{L3}S_{33}+S_{13}^{2}\Gamma_{L3}\Gamma_{L1}\Gamma_{L2}S_{22}+S_{12}^{2}\Gamma_{L2}\Gamma_{L1}\Gamma_{L3}S_{33} \end{array}\right..$$

The Γ*_i_* (*i* = 1, 2, 3) is the reflection coefficient of each port, which is given by

(8) $$\Gamma_{i}=\frac{Z_{L,i}-Z_{0,i}}{Z_{L,i}+Z_{0,i}}$$

where *Z_L,i_* (*i* = 1, 2, 3) is the load impedance of each port, and *Z*_0,*i*_ (*i* = 1, 2, 3) is the characteristic impedance of each port. In this paper, the impedance of Port 1 is matched, i.e., 50 Ω. Therefore, Γ_1_ = 0. *Z_L_*_,2_ and *Z_L_*_,3_ are *Z*_2_ and *Z*_3_, as shown in Figure 3a, respectively. The characteristic impedance of Port 2 or Port 3 is equal to 87.5 Ω.

**Figure 4:**
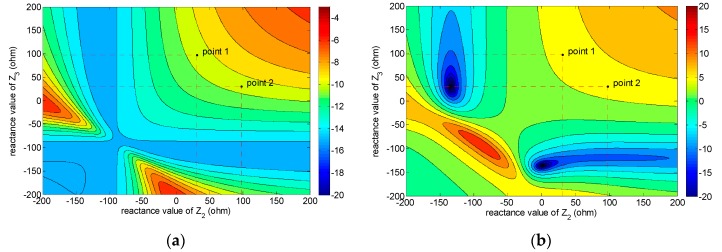
The return loss and gain distribution versus *Z*_2_ and *Z*_3_. (**a**) Return loss. (**b**) Gain at φ = 90°, θ = −20° (The horizontal axis and the vertical axis are the reactance value of load *Z*_2_ and load *Z*_3_).

The simulation results show that the input impedance of Port 2 or Port 3 is always inductive and the real part of the input impedance is so small that it can been neglected. The return loss and the gain at φ = 90°, θ = −20° of the proposed pattern reconfigurable antenna are shown in the contour maps (Figure 4a,b, respectively). In Figure 4, the horizontal axis and the vertical axis are the reactance value of load *Z*_2_ and load *Z*_3_. In order to alleviate the contradiction between the return loss and the gain, the two points in Figure 4 are selected to reconfigure the radiation pattern of the proposed antenna. In these two points, the return loss of the proposed antenna is approximately 10 dB, and the gain reaches approximately 5 dB.

**Figure 5:**
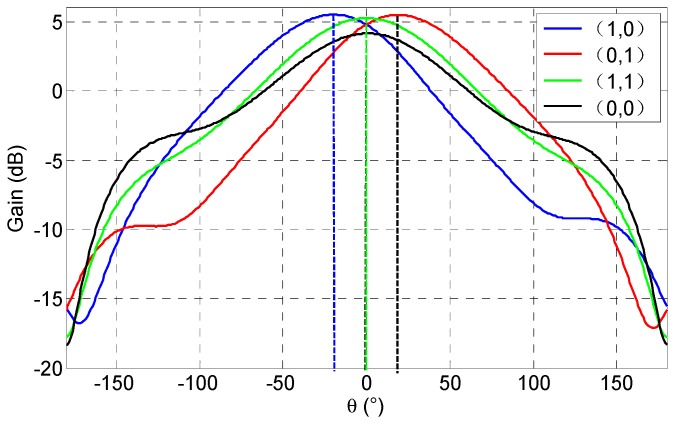
The simulated gain results of the proposed RF MEMS pattern reconfigurable antenna.

The value of load *Z*_2_ and *Z*_3_ can be reconfigured among Point 1 and Point 2 shown in Figure 4 by changing the operating modes. Each RF MEMS switch has two states, i.e., an up state (0) and a down state (1). The input impedance of the Part 2/Part 3 is (0.0808 + *j*1.1140) × 87.5 Ω ≈ (7 + *j*98) Ω in the “0” state, and (0.05 + *j*0.3503) × 87.5 Ω ≈ (4 + *j*31) Ω in the “1” state according to the simulated results, the value of the 87.5 Ω is the characteristic impedance of the port. Thus, the load conditions of the driven patch have four kinds of combination, as shown in Table 4. Fortunately, if the real part of the input impedance (in RF MEMS switch “0” or “1” states) is neglected, the reactance value of the two points in Figure 4 will be reached by changing the RF MEMS switch operating modes among (1,0) and (0,1), i.e., Point 1 corresponding to RF MEMS switch state (1,0) and Point 2 corresponding to (0,1). Therefore, the main lobe direction of the proposed pattern reconfigurable antenna can be steered by changing the RF MEMS switch operating modes. The simulated gain results of the proposed RF MEMS pattern reconfigurable antenna are shown in Figure 5.

## 4. Fabrication, Measurement, and Results

### 4.1. Fabrication

**Figure 6:**
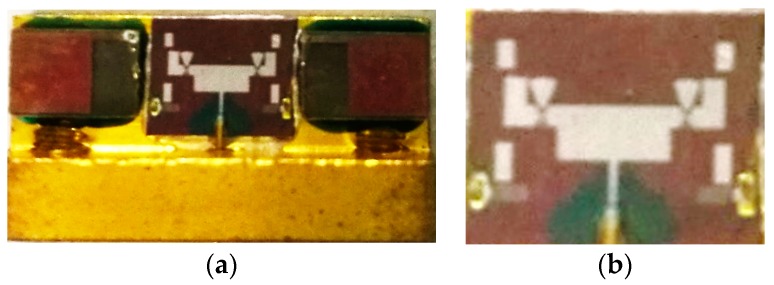
The photograph of the proposed pattern reconfigurable antenna taken by a microscope. (**a**) The antenna with testing holder. (**b**) Close-up of the antenna.

The overall structure of the proposed pattern reconfigurable antenna and RF MEMS switches was fabricated on a high resistivity silicon substrate with a thickness of 400 μm and a dielectric constant of 11.9. The SiO_2_ layer, which acts as an insulating layer, with a thickness of 0.3 μm is formed by thermal oxidation. Then, a 0.2-μm-thick layer of Al is deposited and patterned to define DC bias pads afterward and to form coplanar waveguide (CPW) transmission lines. Next, thin SiAl (approximately 0.05 μm) is patterned by lifting off to form the bias lines after deposition. A Si_3_N_4_ layer with a thickness of 1500 Å is patterned on the top of the electrode and bias lines by a plasma-enhanced chemical vapor deposition (PECVD) process. A 1.5-μm-thick layer of Al, which acts as an anchor, is evaporated. Polyimide as the sacrificial layer was cut down by a chemical mechanical polishing (CMP) process. The beam uses 0.6 μm of SiAl. Finally, the wafer is released in a plasma dryer to avoid the collapse of the membrane. The photographs of the proposed antenna and its close-up are shown in Figure 6.

### 4.2. Measurement and Results

**Figure 7:**
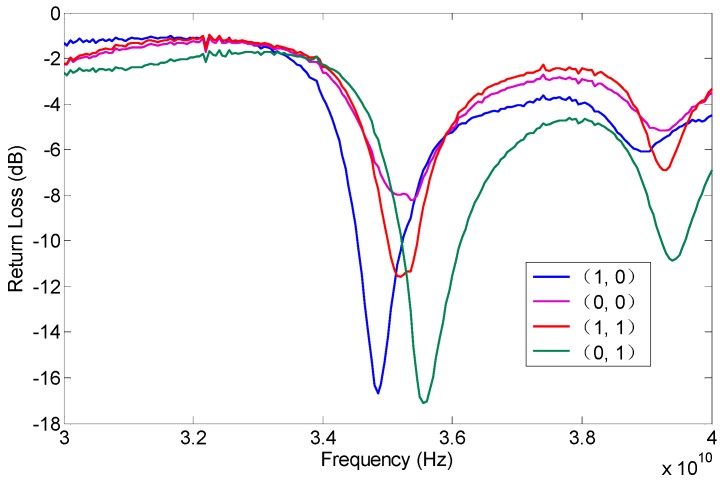
The measured return loss of the proposed pattern reconfigurable antenna in all states of the RF MEMS switches.

Input impedance of all operating modes at the desired frequency of 35 GHz is essential. The return loss of the proposed antenna was measured with the network analyzer Agilent PAN N5442A. The antenna was fed with a 50 Ω microstrip line, and the input impedance of the antenna was transformed by a transformer. The measured return losses of all modes were approximately 10 dB at the desired frequency of 35 GHz, as shown in Figure 7. Thus, the manufactured antenna has acceptable return loss performance.

**Figure 8:**
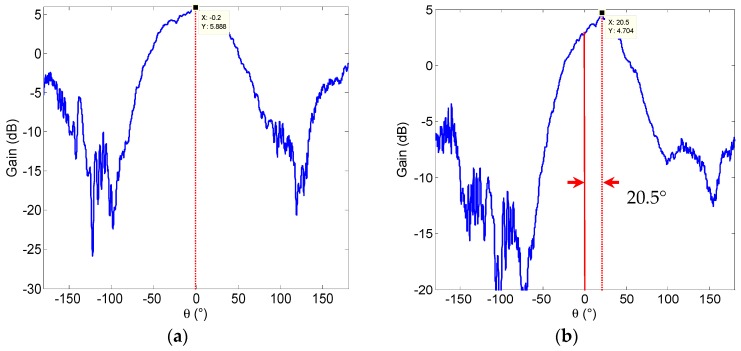

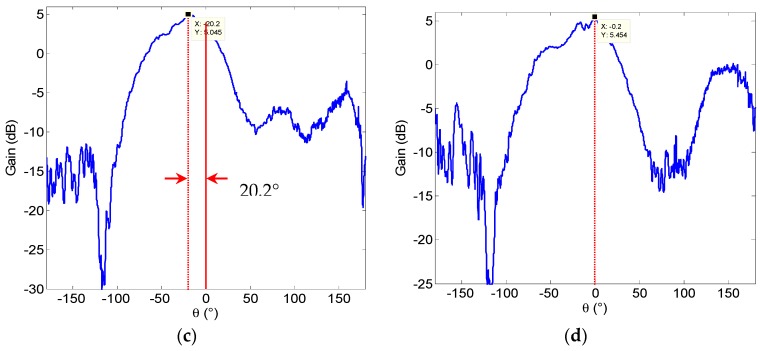
The measured pattern of the proposed antenna using RF MEMS switches. (**a**) In the (0,0) state. (**b**) In the (0,1) state. (**c**) In the (1,0) state. (**d**) In the (1,1) state.

The radiating patterns of the proposed antenna are measured in the microwave chamber. As shown in Figure 8, the proposed pattern reconfigurable antenna can switch the radiating pattern among four operating modes by changing two RF MEMS switches states, and it can obtain three reconfigurable radiating patterns, i.e., left (approximately −20°), middle (approximately 0°), and right (approximately 20°), respectively.

Comparing the measured results, the calculated results, and the simulated results shown in Figure 4 and Figure 5, good agreement is achieved. The measured results in Figure 8 have a back lobe because of the coupling between the antenna patches and the testing holder. The error in fabrication, such as the resolution of lithography, the residual polyimide, the thickness inhomogeneity, the asymmetry between the left RF MEMS switch and the right RF MEMS switch, and the conductor loss are the other primary causes for the discrepancies observed in the measurements. If the manufacturing process is high quality, these discrepancies will be alleviated.

A comparison between the proposed pattern reconfigurable antenna and the available literatures was made, and the comparison results are shown in Table 5. The proposed antenna possesses a compact architecture structure and an acceptable reconfigurable angle range. It can be applied to a 5th-generation (5G) mobile communication and satellite communication system.

## 5. Conclusions

This paper proposes a pattern reconfigurable antenna by employing two RF MEMS switches. By changing the two RF MEMS operating modes, the proposed antenna can switch among three kinds of reconfigurable patterns, namely middle (approximately 0°), left (approximately −20°), and right (approximately 20°). The proposed pattern reconfigurable antenna was analyzed using an active element pattern method and a signal flow diagram. Comparing the measured results with the calculated and simulated results, good agreement was obtained. The proposed pattern reconfigurable antenna can be applied to a 5th-generation (5G) mobile communication and satellite communication system because of its excellent performance.

## Figures and Tables

**Table 1:** The geometry configuration of the proposed antenna.

Symbol	Value (μm)	Symbol	**Value (μm)**	**Symbol**	**Value (μm)**
$W_{du}$	2460	$W_{f}$	383	$W_{1}$	500
$L_{d}$	1000	$W_{au}$	400	$L_{1}$	300
$W_{db}$	2100	$W_{ab}$	580	$W_{2}$	600
$W_{dm}$	130	$L_{al}$	900	$L_{2}$	300
$L_{dm}$	1300	$L_{ar}$	518	-	-

**Table 2:** The reconfigurable radiating pattern main lobe direction and its associated radio frequency micro-electromechanical systems (RF MEMS) switches operating modes.

Mode	Description	Main Lobe Direction (φ = 90°)	Reconfigurable Radiating Pattern
(1,0)	Only left switch in the up state	θ = −20.2	left
(0,0)	Double switches in the up state	θ = −0.2	middle
(1,1)	Double switches in the down state	θ = −0.2	middle
(0,1)	Only right switch in the up state	θ = 20.5	right

**Table 3:** The pivotal size of the designed RF MEMS switch.

Symbol	Value	Symbol	**Value**	**Symbol**	**Value**	**Symbol**	**Value**
$L_{b}$	90 μm	$W_{b}$	340 μm	a	8 μm	δ	46°

**Table 4:** The four kinds of combination for driven patch load conditions.

Switch State	(0,0)	(0,1)	(1,0)	(1,1)
(*Z*_2_, *Z*_3_) Ω	(*j*98, *j*98)	*(j*97.5, *j*30.7)	(*j*30.7, *j*97.5)	(*j*30.7, *j*30.7)

**Table 5:** Performance comparison of the proposed pattern reconfigurable antenna with the literature.

Available Literatures	[4]	[5]	[2]	[18]	This Study
Reconfigurable means	MEMS switches	MEMS switches	PIN diodes	Tunable Graphene Superstrate	MEMS switches
Antenna type	patch	slot-array	Double Layer patch	Double Layer	patch
Operating frequency (GHz)	34.8	30	27.5	30	35
Reconfigurable angles (°)	60	13	45	About 30	40
Block volume (mm^3^)	About 500 × 500 × 2	About 7.112 × 3.556 × 40	5.1 × 5.1 × 1.274	16 × 16 × 10.3	3.7 × 4.4 × 0.4
